# The Fitness of *Pseudomonas aeruginosa* Quorum Sensing Signal Cheats Is Influenced by the Diffusivity of the Environment

**DOI:** 10.1128/mBio.00353-17

**Published:** 2017-05-02

**Authors:** Anne Mund, Stephen P. Diggle, Freya Harrison

**Affiliations:** a Centre for Biomolecular Sciences, School of Life Sciences, University of Nottingham, Nottingham, United Kingdom; b Centre for Mathematics, Technical University of Munich (TUM), Garching, Germany; c School of Biological Sciences, Georgia Institute of Technology, Atlanta, Georgia, USA; d School of Life Sciences, University of Warwick, Coventry, United Kingdom; University of Washington

**Keywords:** *Pseudomonas aeruginosa*, evolution, infectious disease, microbial ecology, opportunistic infections, quorum sensing, virulence

## Abstract

Experiments examining the social dynamics of bacterial quorum sensing (QS) have focused on mutants which do not respond to signals and the role of QS-regulated exoproducts as public goods. The potential for QS signal molecules to themselves be social public goods has received much less attention. Here, we analyze how signal-deficient (*lasI*) mutants of the opportunistic pathogen *Pseudomonas aeruginosa* interact with wild-type cells in an environment where QS is required for growth. We show that when growth requires a “private” intracellular metabolic mechanism activated by the presence of QS signal, *lasI* mutants act as social cheats and outcompete signal-producing wild-type bacteria in mixed cultures, because they can exploit the signals produced by wild-type cells. However, reducing the ability of signal molecules to diffuse through the growth medium results in signal molecules becoming less accessible to mutants, leading to reduced cheating. Our results indicate that QS signal molecules can be considered social public goods in a way that has been previously described for other exoproducts but that spatial structuring of populations reduces exploitation by noncooperative signal cheats.

## IMPORTANCE

Bacteria communicate via signaling molecules to regulate the expression of a whole range of genes. This process, termed quorum sensing (QS), moderates bacterial metabolism under many environmental conditions, from soil and water (where QS-regulated genes influence nutrient cycling) to animal hosts (where QS-regulated genes determine pathogen virulence). Understanding the ecology of QS could therefore yield vital clues to how we might modify bacterial behavior for environmental or clinical gains. Here, we demonstrate that QS signals act as shareable public goods. This means that their evolution, and therefore population-level responses to interference with QS, will be constrained by population structure. Further, we show that environmental structure (constraints on signal diffusion) alters the accessibility of QS signals and demonstrates that we need to consider population and environmental structure to help us further our understanding of QS signaling systems.

## INTRODUCTION

Bacterial quorum sensing (QS) is a cell-to-cell signaling mechanism that coordinates a range of behaviors at the population level (1, 2). QS facilitates density-dependent production of extracellular molecules, including nutrient-scavenging enzymes and virulence factors. These molecules have been termed “public goods” because their benefits can be shared by all cells in the local population (3–6). Because these QS-regulated exoproducts are metabolically costly for cells to produce, QS can also be exploited by noncooperating “cheats”: cells that do not respond to QS signals and so pay no costs but which exploit wild-type populations because they benefit from the public goods produced by wild-type neighboring cells (4–7). Experiments into the social dynamics of QS have traditionally focused on these “signal-blind” mutants, and a number of studies have shown that such mutants can arise in laboratory cultures and during infections (8–14). Under various laboratory conditions and in *in vivo* infection models, they have been shown to act as social cheats (6, 15–17).

However, little attention has been paid to whether QS signals themselves can act as exploitable public goods, despite there being a metabolic cost associated with the production of QS signals (even in the absence of downstream responses [18, 19]). Previous experiments have shown that signal-negative mutants can act as cheats, but these were conducted under conditions where QS-dependent exoproducts enhance growth: these studies therefore do not separate the fitness effects of producing from those of responding to signal (5, 6). Here, we analyze how signal-negative (*lasI*) mutants of the opportunistic pathogen *Pseudomonas aeruginosa* socially interact with wild-type cells in an environment where growth requires the cells to have a functional QS system but where the fitness benefits of QS are “private” to individual cells. *P. aeruginosa* regulates the production of many virulence factors through two *N*-acylhomoserine lactone (AHL)-based QS systems. These systems, termed the *las* and *rhl* systems, produce and respond to the signals *N*-(3-oxododecanoyl)-l-homoserine lactone (3O-C_12_-HSL) and *N*-butanoyl-l-homoserine lactone (C_4_-HSL), respectively (1, 20). We conducted our experiments in a growth medium containing adenosine as a carbon source. Adenosine is deaminated to form inosine, which is degraded inside the cell by a nucleoside hydrolase (Nuh) to hypoxanthine plus ribose; hypoxanthine is then metabolized to produce glyoxylate plus urea (21). QS is crucial for growth in this medium because the *las* system (through the regulator LasR) positively regulates Nuh. Because Nuh acts intracellularly, any loss of fitness due to mutation of the signal gene *lasI* will be directly due to the lack of signal—not to any downstream effect on the production of extracellular enzymes. We demonstrate that, when provided with adenosine as a carbon source, *lasI* mutants act as cheats: they grow poorly in monoculture but have a higher relative fitness than the signal-producing wild type in mixed cultures. In contrast, *lasR* mutants, which cannot regulate Nuh in the presence of signal, do not gain any fitness benefits in mixed culture with wild-type cells.

In contrast to experiments performed in well-mixed liquid medium in test tubes, interactions between bacterial cells in natural environments (including infections) are affected by spatial assortment and structuring (22–24). This affects how behaviors evolve (25–29). We tested how simple spatial structuring, through the addition of agar to the growth medium, alters QS signal diffusion and the social dynamics of wild-type and *lasI* mutant cells. Consistent with work on other bacterial public goods (28), we demonstrate that the ability of *lasI* mutants to cheat is significantly reduced in structured populations. These results have implications for understanding how and why bacterial signaling evolves and the likely evolutionary fate of different types of QS mutants under varied environmental conditions (30).

## RESULTS

### *las* mutants grow poorly in an environment where QS is required for growth.

**FIG 1:**
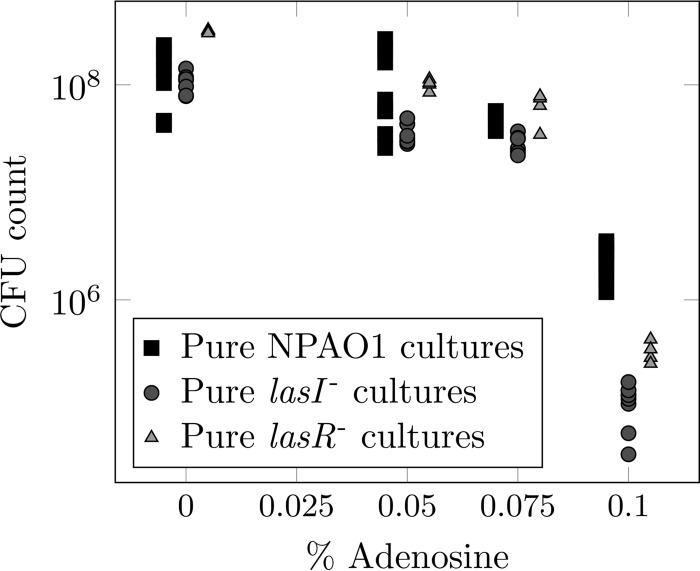
Population density (CFU) after 48 h of growth in quorum sensing medium with various ratios of Casamino Acids to adenosine supplied to a total concentration of 0.1% (wt/vol) carbon source. Squares, individual wild-type cultures; circles, *lasI* mutant cultures; triangles, *lasR* mutant cultures.

Previous work has shown that *las* mutants grow poorly in medium where QS is required for growth (5, 7, 31). We first confirmed that both *lasI* and *lasR* mutants were reduced in fitness in the specific medium that we chose for our experiments. We grew PAO1 and each mutant in a minimal medium base containing 0.1% (wt/vol) carbon source. The carbon source was composed of Casamino Acids (CAA; available for use by all cells, regardless of genotype) and adenosine (which can be metabolized only when QS is functional in cells), in various ratios. As the relative amount of adenosine increased and cells were increasingly dependent on QS, the total cell density after 48 h was reduced, and this effect was more pronounced in *lasI* mutant monocultures than in wild-type monocultures (Fig. 1) (when all the available carbon was supplied as adenosine, wild-type cultures grew to 4.6% of the density that they attained when all carbon was supplied as CAA; for *las* mutants, this value was 0.1 to 0.2%). When all the available carbon was supplied as adenosine, *lasI* mutant monocultures grew to approximately 7% of the density of wild-type monocultures.

### Signal-negative *lasI* mutants act as social cheats in adenosine-based growth medium, but signal-blind *lasR* mutants do not.

**FIG 2:**
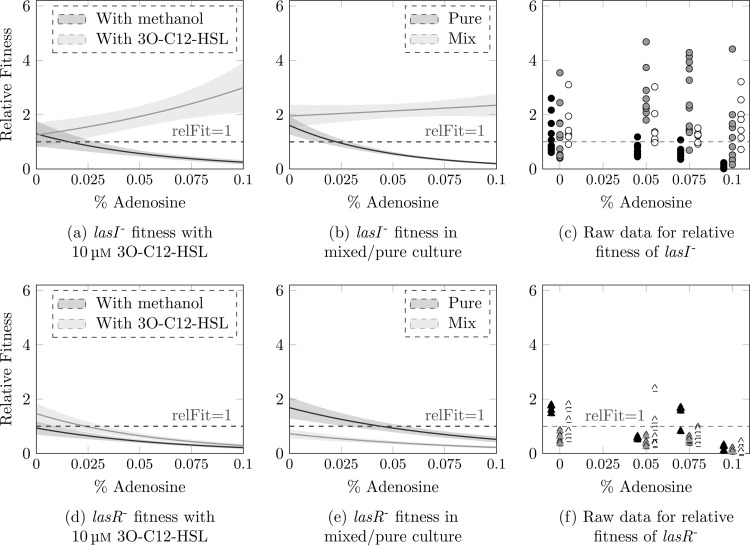
Results of fitting generalized linear models to relative fitness data from experiments with *lasI* (a and b) and *lasR* (d and e) mutants. Lines show fitted models; shaded areas denote standard deviations. Relative fitness is compared between pure cultures of each mutant with added 3O-C_12_-HSL or with a solvent-only control (a and d) and between pure cultures and mixed culture with wild-type bacteria (b and e). Raw data for these experiments are shown in panels c and f (black symbols, fitness in pure culture [solvent-only control]; white symbols, fitness in pure culture supplemented with 10 µM 3O-C_12_-HSL; gray symbols, fitness in a 1:1 mixture with wild type).

We next tested whether adding purified 3O-C_12_-HSL, or coculture with signal-producing wild-type bacteria, could restore the growth of *lasI* mutants. We calculated the fitness of *lasI* mutants relative to the wild type (i) in pure culture with or without exogenous 3O-C_12_-HSL and (ii) in 1:1 coculture with wild-type PAO1. Experiments were conducted in quorum sensing medium with various ratios of CAA and adenosine as described above, and cultures were grown for 48 h. A relative fitness of 1 signifies similar growth of mutant and wild-type bacteria, while values of <1 reflect relatively poorer growth of the mutant and values of >1 reflect better growth of the mutant. Figure 2 shows raw data and fitted models describing how the relative fitness of *lasI* (Fig. 2a, b, and c) and *lasR* (Fig. 2d, e, and f) mutants is affected by culture conditions.

Pure *lasI* mutant cultures became progressively less fit than the wild type as access to carbon depended more on quorum sensing (negative correlation between relative fitness and percent adenosine: coefficient, −21.6; *P* < 0.001). However, when 10 µM exogenous 3O-C_12_-HSL was supplied, *lasI* mutants surpassed the wild type in growth (positive correlation between relative fitness and percent adenosine: coefficient, 8.74, *P* = 0.003) (Fig. 2a and c) This result is consistent with previous work demonstrating a cost to 3O-C_12_ production (18). As predicted, this ability of *lasI* mutants to use exogenous signal, combined with the cost of signal production to the wild type, means that *lasI* mutants grown in coculture with wild-type bacteria act as social cheats: the average relative fitness was consistently >1 and did not decline as percent adenosine increased (coefficient, 1.8; *P* = 0.88) (Fig. 2b and c). There was, however, a slight drop in relative fitness when all carbon was supplied as adenosine (Fig. 2c). This is most likely attributable to the wild-type bacteria growing more slowly and taking longer to fully switch on QS responses. Both wild-type growth (Fig. 1) and the pool of available signal (see Fig. S2 in the supplemental material) are reduced under this condition, leaving less opportunity for exploitation by cheats.

*Post hoc* comparisons confirmed that *lasI* mutant relative fitness was significantly increased in mixed populations versus pure cultures in all media containing adenosine (*t* tests, *P* < 0.01). When all carbon was supplied as CAA and signal was not required, there was no significant effect of pure versus mixed culture on fitness (*P* > 0.95) Taken together with the fact that mixed cultures grew to a lower density than the wild-type cultures (Fig. S3), these results indicate that *lasI* mutants have an increased fitness when grown in the presence of wild-type bacteria under conditions requiring social interaction, while in turn decreasing wild-type fitness. Theory predicts that when population growth is strongly dependent on cooperation, cheats should be under negative frequency-dependent selection (they should have a greater fitness advantage when rarer [26, 32]). Consistent with this prediction, the relative fitness of the *lasI* mutant in intermediate adenosine/CAA ratios was negatively correlated with its initial frequency in the population (Fig. S4).

To ensure that the effect described above was due to the social dynamics of signal production, and not to the well-documented social dynamics of downstream exoproducts, this experiment was repeated using a *lasR* mutant. *lasR* mutants are unable to respond to 3O-C_12_-HSL and should therefore not be able to derive fitness benefits from exogenous signal in our setup. We found the same negative correlations between percent adenosine and CFU (Fig. 1) and percent adenosine and fitness relative to the wild type (Fig. 2d and f) (coefficient, −12.8; *P* = 0.001) as with the *lasI* mutant for *lasR* mutant monocultures. Crucially, *lasR* mutant relative fitness was not rescued by adding 3O-C_12_-HSL or by coculture with the wild type (coefficient, −10.7; *P* < 0.001) (Fig. 2d, e, and f), i.e., these mutants could not exploit wild-type bacteria.

### Slowing signal diffusion reduces *lasI* mutant cheating.

**FIG 3:**
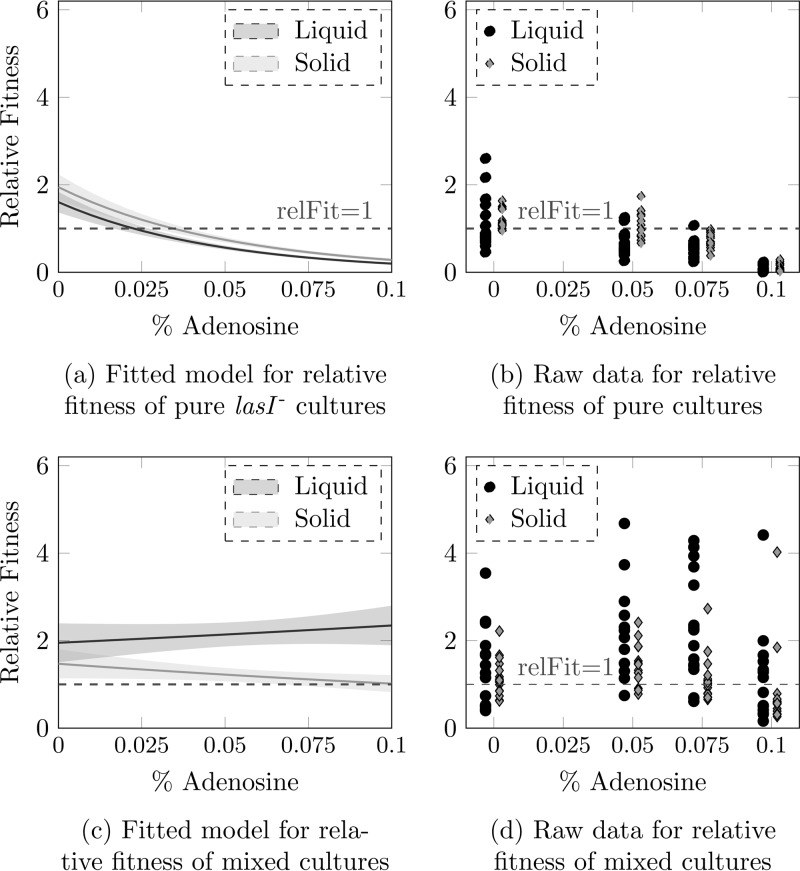
Comparison of *lasI* mutant relative fitness in pure culture (a and b) or mixed culture with the wild type (c and d). Panels a and c show the results of fitting generalized linear models to relative fitness data from experiments in liquid versus agar-supplemented medium. Lines show fitted models; shaded areas denote standard deviations. Raw data are shown in panels b and d (black circles, liquid medium; gray diamonds, agar-supplemented medium).

As a last step, we tested whether impeding the diffusion of signal molecules would make the *lasI* mutant less effective as a cheat. Reduced diffusion was achieved by adding agar to solidify the growth medium (Fig. S1). *lasI* mutant monocultures showed comparable declines in fitness in liquid and solid media (Fig. 3a and b) (analysis of variance [ANOVA], liquid/solid *F*^1,124^ = 6.13, *P* = 0.01; adenosine *F*^1,123^ = 117.58, *P* < 0.001; interaction *F*^1,122^ = 0.1658, *P* = 0.68). In mixed cultures, the relative fitness of the *lasI* mutant was positively correlated with the percentage of carbon available as adenosine in liquid cultures, as expected under cheating, but when the medium was solidified, *lasI* mutant relative fitness actually showed a modest negative correlation with percent adenosine (Fig. 3c and d) (ANOVA, liquid/solid *F*^1,123^ = 11.1324, *P* = 0.001; adenosine *F*^1,124^ = 0.0236, *P* = 0.88; interaction *F*^1,122^ = 0.9760, *P* = 0.33). This demonstrates that there is less opportunity for cheating in an environment where signal molecules cannot diffuse freely.

10.1128/mBio.00353-17.1 FIG S1  Supplementing growth medium with agar retards diffusion of 3O-C_12_-HSL. (a) Expression of a 3O-C_12_-HSL-dependent luminescent reporter construct is delayed and reduced when signal-negative reporter bacteria are separated from a reservoir or purified signal by an agar barrier. Lines and shading show means and standard deviations for 15 replicates. (b) The experimental setup, as described in Materials and Methods, is easily visualized by replicating the experiment on a larger scale and adding red food coloring in place of bacterial signal. Download FIG S1, PDF file, 2.8 MB. Copyright © 2017 Mund et al. 2017 Mund et al. This content is distributed under the terms of the Creative Commons Attribution 4.0 International license.

10.1128/mBio.00353-17.2 FIG S2  Measurements of 3O-C_12_-HSL concentration in wild-type supernatant after 48 h. (a) The total concentration of 3O-C_12_-HSL declines with increasing adenosine. (b) After dividing the signal concentration by the CFU of the producing culture, one observes the reverse trend—concentration of 3O-C_12_-HSL per CFU increases with adenosine level and reaches its maximum in pure adenosine. Download FIG S2, PDF file, 0.1 MB. Copyright © 2017 Mund et al. 2017 Mund et al. This content is distributed under the terms of the Creative Commons Attribution 4.0 International license.

10.1128/mBio.00353-17.3 FIG S3  Population density (CFU) after 48 h of growth in quorum sensing medium, Blue circles, individual wild-type cultures; yellow circles, *lasI* mutant and wild-type mixed cultures; red circles, *lasI* cultures. Download FIG S3, PDF file, 0.05 MB. Copyright © 2017 Mund et al. 2017 Mund et al. This content is distributed under the terms of the Creative Commons Attribution 4.0 International license.

10.1128/mBio.00353-17.4 FIG S4  Relative fitness of *lasI* mutants after 48-h growth in medium containing intermediate levels of adenosine is negatively correlated with their initial frequency in the population (coefficient, −0.69; *P* < 0.001). Panel a shows the result of fitting a linear model to relative fitness data from experiments with different starting frequencies of *lasI* in 0.05% and 0.075% adenosine. Lines show fitted models; shaded areas denote standard deviations. Raw data for these experiments are shown in panel b (black circles, 0.05% adenosine; gray diamonds, 0.075% adenosine). Download FIG S4, PDF file, 0.1 MB. Copyright © 2017 Mund et al. 2017 Mund et al. This content is distributed under the terms of the Creative Commons Attribution 4.0 International license.

## DISCUSSION

While there has been some discussion of QS signals as public goods (e.g., reference 33), most published work on the social evolution of QS focuses on signal-blind mutants and the benefits of cheating on the production of QS-regulated exoproducts (5, 7, 14, 34). Here, we provide the first direct evidence that, in addition to regulating the production of public goods, QS signal molecules are themselves capable of acting as public goods. Social cheating in the context of QS can therefore take multiple forms, depending on the environmental circumstances in which bacteria find themselves. Previous research had shown that (i) signal-blind mutants can be cheats when growth depends on the production of QS-dependent extracellular enzymes and (ii) cheating by signal-blind mutants can be constrained when some “private” QS-controlled processes contribute to growth (31). Following recent confirmation that QS signals are costly to make (18), we now add two new perspectives to the social evolution of QS: (iii) that signal-negative mutants can be cheats when growth depends entirely on private goods, regardless of any downstream effects on exoenzyme production, and (iv) that this signal cheating can occur only when the environment permits sufficient diffusion of signal molecules.

A *lasI* mutant grew poorly in monoculture, but growth could be rescued either by adding purified 3O-C_12_-HSL signal or by coculture with wild-type, signal-producing bacteria. In cocultures, the fitness of *lasI* mutants relative to the wild type increased as we forced the bacteria to rely more on adenosine for carbon. As the population became more reliant on QS, *lasI* mutants gained a greater fitness payoff from exploiting costly, diffusible signal produced by the wild type. *lasR* mutants did not gain a similar advantage from coculture with the wild type in adenosine medium. These signal-blind mutants can take up signals but cannot respond to them and so cannot switch on expression of the *nuh* hydrolase required for growth on adenosine (21, 31). The contrasting results for the two different QS mutants confirm that, in this environment, the QS signal itself acts as a public good.

Adding agar to the growth medium lowered both the diffusion of signal molecules and the relative fitness of cheats in mixed culture. Thus, adding simple spatial structuring into our system had a significant impact on the ability of signal-negative mutants to cheat on the wild type. There was no effect of structuring on relative fitness in pure cultures: even though agar enhanced the overall growth rate of the bacteria, the basic costs and benefits of signaling remained the same in liquid and solid media. This final observation is consistent with work on other bacterial public goods (28). We thus predict that the evolution of QS signaling strategies will be influenced by population genetic and spatial structure and that signal-negative cheats might rise to appreciable frequencies only in environments where signals diffuse freely. For example, the thick, adhesive mucus and bacterial biofilm polymers that block the airways of cystic fibrosis patients with chronic lung infection may partially protect producers from cheating by signal-negative mutants (24). Spatial dynamics play a huge role in the real-life ecology of environmentally and clinically important microbial ecosystems and are therefore of considerable interest to microbiologists investigating the roles of bacteria in processes as diverse as geochemical cycling, soil health, fouling, and infection (35–37).

Our work opens up new avenues for exploring how, when, and why bacterial signaling evolves in different environments and why we find a variety of QS mutant genotypes and phenotypes in natural environments (8–14, 38, 39). *P. aeruginosa* clones carrying mutations in the *lasI* (3O-C_12_-HSL) and *rhll* (C_4_-HSL) QS signals have been isolated from chronically infected cystic fibrosis patients and mechanically ventilated hospital patients (8–11). Given what we are now learning about the evolution of traits such as QS and how spatial structure changes the evolutionary dynamics, we suggest that there is a need to carefully consider the experimental design of *in vitro* experiments to increase their relevance to actual infections (40). To be forewarned is to be forearmed: a more accurate understanding of microbial ecology and evolution, gained from more realistic lab experiments, will be a vital weapon in the fight against infection.

## MATERIALS AND METHODS

### Bacterial strains.

The strains used were the wild-type *Pseudomonas aeruginosa* laboratory strain PAO1 and isogenic mutants created via insertion of a gentamicin resistance gene in the QS genes *lasI* (PAO1 *lasI*::Gm; referred to as *lasI* mutant [41]) and *lasR* (PAO1 *lasR*::Gm; referred to as *lasR* mutant [16]). To test 3O-C_12_-HSL diffusion in different media, we used a reporter strain of the *lasI* mutant. This contains a chromosomal *luxCDABE* fusion to the promoter of the *lasB* gene, which encodes the QS-dependent protease LasB (PAO1 p*lasB*::*lux* [41]).

### Growth conditions.

Quorum sensing medium (QSM) was modified from previous studies (5, 34, 42). QSM consisted of OS minimal medium [7.01 g Na_2_HPO_4_, 6.8 g KH_2_PO_4_, 1.19 g MgSO_4_⋅7H_2_O, 1 g (NH_4_)_2_SO_4_, 88 mg CaCl_2_⋅2H_2_O, 2 mg FeSO_4_⋅7H_2_O, 0.2 mg (NH_4_)_6_Mo_7_O_24_⋅4H_2_O, 1 ml Hutner’s “Metals 44,” per liter] (43) supplemented with 0.1% (wt/vol) carbon sources as a mix of Casamino Acids (CAA) and adenosine. The medium was filter sterilized. The exact ratio of CAA and adenosine was varied as detailed in Results. Liquid culture experiments were conducted in 24-well plates with a volume of 2 ml of medium. Cultures were incubated overnight in LB medium at 37°C on an orbital shaker and standardized to an optical density at 600 nm (OD_600_) of 0.8 to 0.9; 2 µl of pure or mixed inoculum was added to each experimental culture. The starting frequency of the mutant was determined by diluting and plating the starter cultures to determine the number of CFU of each genotype. Experimental cultures in QSM were incubated at 37°C with orbital shaking for 24 h or 48 h. After that time, cultures were diluted and replica plated on LB and LB plus gentamicin (25 μg/ml) agar to enumerate the CFU of PAO1 and *lasI* or *lasR* mutants in mixed culture. Experiments in solid media were conducted in QSM plus 2% (wt/vol) agar in 1-ml volumes in 48-well plates. Inoculation and culture conditions were otherwise identical to experiments in liquid medium. To break up agar prior to dilution and plating, the solid 1-ml agar cubes were retrieved from the plate and divided into thirds with a sterile metal spatula; each third was placed in a screw-cap tube containing 500 μl phosphate-buffered saline and 6 metal beads (Cambio) and homogenized using a FastPrep-24 5G bead beater (MP Biomedicals).

To test whether the *lasI* mutant was under negative frequency-dependent selection in medium where cheating could occur in the above-described experiments, mixed populations containing *lasI* mutants at initial frequencies of 1%, 10%, 40%, and 60%, as well as pure wild-type and *lasI* mutant cultures, were inoculated into 2 ml liquid QSM containing 0.05% (wt/vol) adenosine plus 0.05% (wt/vol) CAA and 2 ml liquid QSM containing 0.075% (wt/vol) adenosine plus 0.025% (wt/vol) CAA in 24-well plates for 5-fold replication. Cultures were grown for 48 h on an orbital shaker and replica plated as described above.

### Measures of signal concentration.

To measure the concentration of QS signal (*N*-3-oxododecanoyl-l-homoserine lactone [3O-C_12_-HSL]) present in 48-h cultures, 100 µl of each culture supernatant was mixed with 100 µl of a log-phase culture of a luminescent *Escherichia coli* bioreporter (pSB1075 [44]) in the wells of a 96-well plate. This mixture was incubated for 4 h in a Tecan multimode plate reader, and luminescence and OD_600_ were recorded at 15-min intervals. To estimate the 3O-C_12_-HSL concentration, the luminescence of experimental samples was compared with a calibration curve constructed using QSM supplemented with known concentrations of purified 3O-C_12_-HSL.

### Assaying the effect of agar on QS signal diffusion.

Agar has been successfully used to retard the diffusion of other bacterial exoproducts (28). To verify that agar affects 3O-C_12_-HSL diffusion in QSM and to determine the optimal agar concentration to use in further experiments, we devised a “sandwich experiment” in which a population of bacteria that switch on a luminescent reporter gene in response to QS signal but which cannot themselves produce signal were separated from a reservoir of purified signal by a layer of agar-supplemented medium. By measuring the time to expression of the luminescent reporter, we can assess the extent to which the agar barrier delays diffusion of the signal from the reservoir to the reporter population. An 0.1-ml amount of LB supplemented with 0.5% (wt/vol) agar and containing 0.5 µM purified 3O-C_12_-HSL was added to the wells of a 48-well plate and allowed to solidify. A second layer of 0.8 ml LB supplemented with 1, 2, 3, or 4% (wt/vol) agar was then added on top of the signal-containing layer. Each agar concentration was replicated in 6 wells. This layer was allowed to solidify, and a final layer of LB containing 0.5% (wt/vol) agar and the reporter PAO1 *lasI* mutant p*lasB*::*lux* (overnight culture at OD_600_ of 0.2) was added. The plate was incubated in a Tecan multimode reader for 8 h, and luminescence was read at 10-min intervals. As shown in Fig. S1 in the supplemental material, an increasing agar concentration progressively delayed and reduced expression of luminescence. In order to check if higher luminescence was due to increased bacterial numbers, bacteria were retrieved and CFU were counted by plating. Median CFU was similar when 1% or 2% agar was used (approximately 1.4 × 10^7^) but decreased by 30% when more agar was added (to approximately 1 × 10^7^). It was difficult to determine whether this was due to agar retarding growth at high concentrations or simply due to the increased difficulty of thoroughly homogenizing medium rich in agar. One percent agar was therefore chosen for use in further experiments.

### Statistical analysis.

Relative fitness of mutants, *v*, was calculated as *x*_2_(1 − *x*_1_)/*x*_1_(1 − *x*_2_), where *x*_1_ is the starting frequency of the mutant and *x*_2_ is the end frequency. It follows from the definition that a relative fitness of <1 signifies a decrease in mutant frequency, while a relative fitness of >1 signifies an increase in mutant frequency. To calculate relative fitness of the mutant in pure culture, mutant and wild-type monocultures were randomly paired. Statistical analysis of the results was conducted in R 3.2.3 (45) using generalized linear models assuming an underlying gamma distribution, with adenosine treated as a continuous variable and block and treatment (liquid-solid medium) fitted as factors. Raw data for all analyses reported are supplied in the supplemental material (Data Set S1).

10.1128/mBio.00353-17.5 DATA SET S1  Data for all analyses reported in the text (Mundetal_rawdata.xlsx). Download DATA SET S1, XLSX file, 0.02 MB. Copyright © 2017 Mund et al. 2017 Mund et al. This content is distributed under the terms of the Creative Commons Attribution 4.0 International license.
