# Erratum for Mund et al., “The Fitness of *Pseudomonas aeruginosa* Quorum Sensing Signal Cheats Is Influenced by the Diffusivity of the Environment”

**DOI:** 10.1128/mBio.00816-17

**Published:** 2017-06-13

**Authors:** Anne Mund, Stephen P. Diggle, Freya Harrison

**Affiliations:** a Centre for Biomolecular Sciences, School of Life Sciences, University of Nottingham, Nottingham, United Kingdom; b Centre for Mathematics, Technical University of Munich (TUM), Garching, Germany; c School of Biological Sciences, Georgia Institute of Technology, Atlanta, Georgia, USA; d School of Life Sciences, University of Warwick, Coventry, United Kingdom

## ERRATUM

Volume 8, no. 3, e00353-17, 2017, https://doi.org/10.1128/mBio.00353-17. The end of the first paragraph in the Acknowledgments reads “and the Technical University of Munich (TUM) in the framework of the Open Access Publishing Program.” This is incorrect and should instead read “and RCUK (open access charges).”

