# Supplementary figures and images for: The Fitness of Pseudomonas aeruginosa Quorum Sensing Signal Cheats Is Influenced by the Diffusivity of the Environment
Source: mBio. 2017 May 2;8(3):e00353-17. doi: 10.1128/mBio.00353-17 (PMC5414003; doi:10.1128/mBio.00353-17)

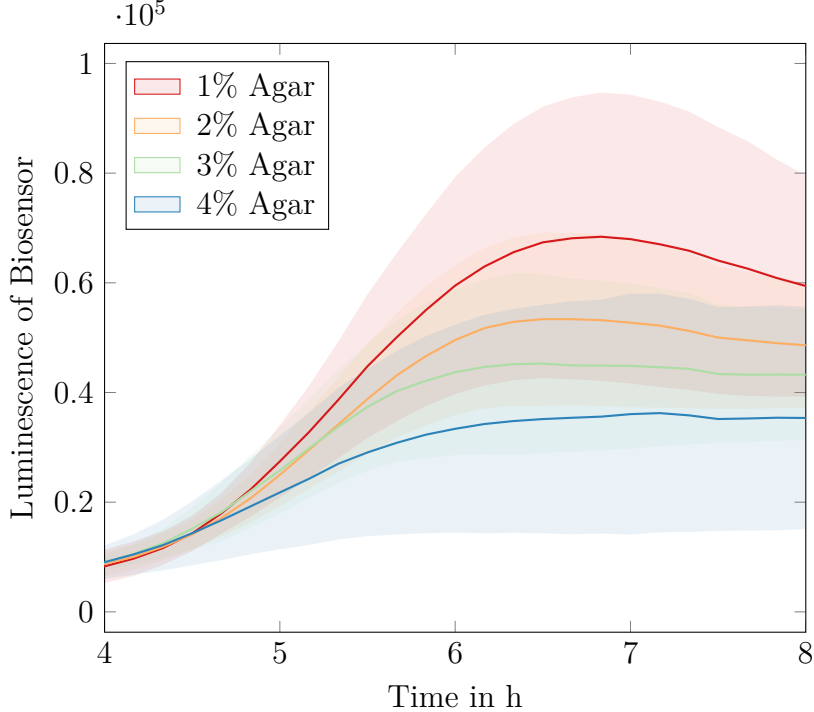

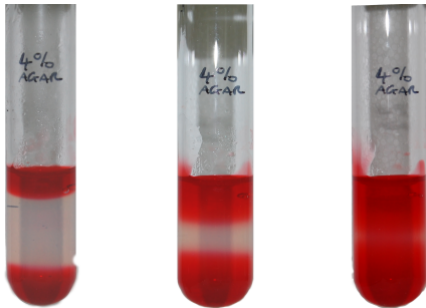

time →

Supplement: FIG S1 [file mbo002173272sf1.pdf]

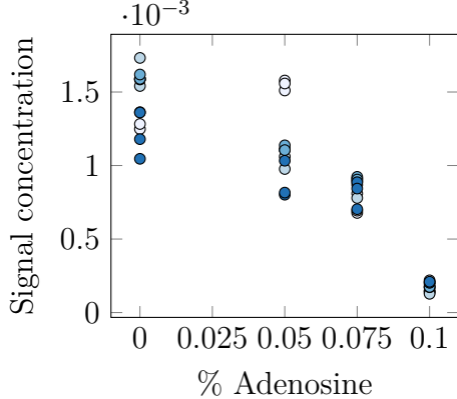

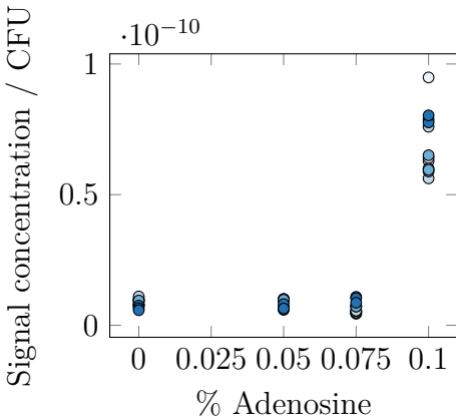

Supplement: FIG S2 [file mbo002173272sf2.pdf]

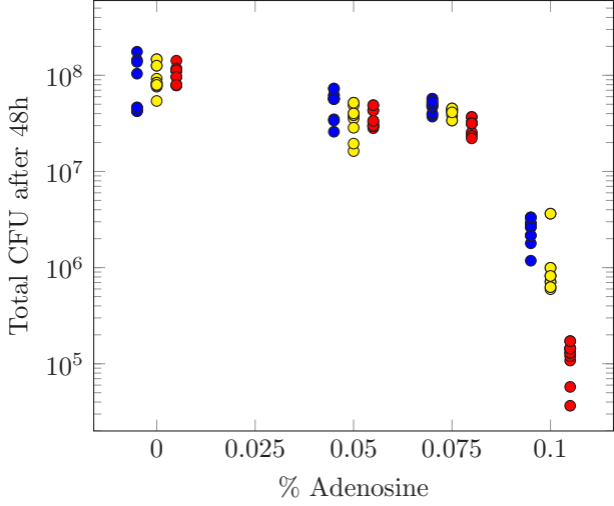

Supplement: FIG S3 [file mbo002173272sf3.pdf]
