# Supplementary material for: The Fitness of Pseudomonas aeruginosa Quorum Sensing Signal Cheats Is Influenced by the Diffusivity of the Environment
Source: mBio. 2017 May 2;8(3):e00353-17. doi: 10.1128/mBio.00353-17 (PMC5414003; doi:10.1128/mBio.00353-17)
Supplement: FIG S4 [file mbo002173272sf4.pdf]

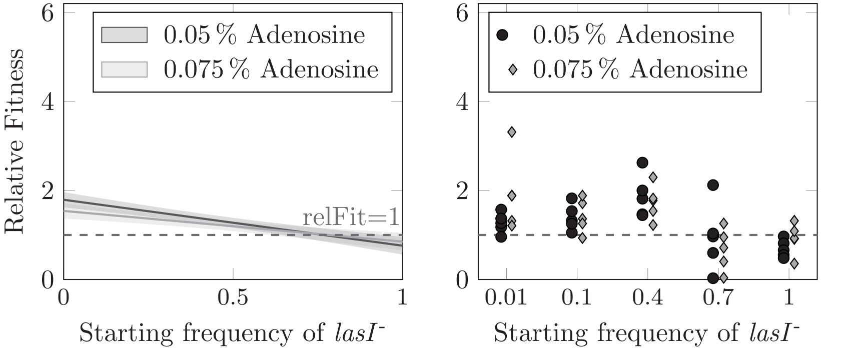

(a) Fitted model for relative fitness of cultures with different start frequencies of *lasI*-

(b) Raw data for relative fitness of different start frequencies
